# *Plasmodium falciparum* Parasitemia Does Not Diminish Neutralizing Antibody Responses After mRNA COVID-19 Booster Vaccination in HIV-infected Adults

**DOI:** 10.1093/infdis/jiaf398

**Published:** 2025-08-02

**Authors:** Taraz Samandari, Millicent Achola, Jack N Hutter, Grace Mboya, Walter Otieno, Jia Jin Kee, Yunda Huang, John J Aponte, Christian F Ockenhouse, Cynthia K Lee, Laura Polakowski, Margaret Yacovone, Asa Tapley, Sufia Dadabhai, Nonhlanhla N Mkhize, Haajira Kaldine, Sinethemba Bhebhe, Penny L Moore, John Hural, Nigel Garrett, James G Kublin

**Affiliations:** COVID-19 Prevention Network, Seattle, Washington, USA; U.S. Army Medical Research Directorate-Africa, Kisumu, Kenya; U.S. Army Medical Research Directorate-Africa, Kisumu, Kenya; Kenya Medical Research Institute, Centre for Global Health Research, Kisumu, Kenya; U.S. Army Medical Research Directorate-Africa, Kisumu, Kenya; Vaccine and Infectious Diseases Division, Fred Hutchinson Cancer Center, Seattle, Washington, USA; Vaccine and Infectious Diseases Division, Fred Hutchinson Cancer Center, Seattle, Washington, USA; Department of Global Health, University of Washington, Seattle, Washtington, USA; PATH Center for Vaccine Innovation and Access, Geneva, Switzerland; PATH Center for Vaccine Innovation and Access, Washington, District of Columbia, USA; PATH Center for Vaccine Innovation and Access, Washington, District of Columbia, USA; National Institute of Allergy and Infectious Diseases, National Institutes of Health, Bethesda, USA; National Institute of Allergy and Infectious Diseases, National Institutes of Health, Bethesda, USA; Vaccine and Infectious Diseases Division, Fred Hutchinson Cancer Center, Seattle, Washington, USA; Division of Allergy and Infectious Diseases, Department of Medicine, University of Washington, Seattle, Washington, USA; Department of Medicine, University of Cape Town, Cape Town, South Africa; Johns Hopkins Bloomberg School of Public Health, Blantyre, Malawi; National Institute for Communicable Diseases of the National Health Laboratory Services, Johannesburg, South Africa; SA MRC Antibody Immunity Research Unit, School of Pathology, Faculty of Health Sciences, University of the Witwatersrand, Johannesburg, South Africa; National Institute for Communicable Diseases of the National Health Laboratory Services, Johannesburg, South Africa; SA MRC Antibody Immunity Research Unit, School of Pathology, Faculty of Health Sciences, University of the Witwatersrand, Johannesburg, South Africa; National Institute for Communicable Diseases of the National Health Laboratory Services, Johannesburg, South Africa; SA MRC Antibody Immunity Research Unit, School of Pathology, Faculty of Health Sciences, University of the Witwatersrand, Johannesburg, South Africa; National Institute for Communicable Diseases of the National Health Laboratory Services, Johannesburg, South Africa; SA MRC Antibody Immunity Research Unit, School of Pathology, Faculty of Health Sciences, University of the Witwatersrand, Johannesburg, South Africa; Vaccine and Infectious Diseases Division, Fred Hutchinson Cancer Center, Seattle, Washington, USA; Desmond Tutu HIV Centre, University of Cape Town, Cape Town, South Africa; Centre for the AIDS Programme of Research in South Africa, University of KwaZulu-Natal, Durban, South Africa; Discipline of Public Health Medicine, School of Nursing and Public Health, University of KwaZulu-Natal, Durban, South Africa; Vaccine and Infectious Diseases Division, Fred Hutchinson Cancer Center, Seattle, Washington, USA

**Keywords:** mRNA vaccine, malaria, HIV, SARS-CoV-2, immunogenicity

## Abstract

mRNA vaccines have emerged as powerful tools for the prevention of infectious diseases, but subclinical malaria may reduce vaccine immunogenicity. We evaluated neutralizing antibody responses in asymptomatic HIV-infected adults with and without polymerase chain reaction-confirmed *Plasmodium falciparum* who received either monovalent mRNA-1273 or bivalent mRNA-1273.222 (WA-1 and BA.4/5) booster vaccines. In previous studies, a 50% pseudovirus inhibitory dose neutralizing antibody (ID50) titer of 1000 correlated with 96% efficacy in preventing COVID-19. We observed ID50 geometric mean titers >22 000 in both parasitemic and nonparasitemic participants 1 month after boosting. We conclude that COVID-19 mRNA vaccine antibody responses are unimpaired by concurrent asymptomatic parasitemia.

## Teaser

COVID-19 booster mRNA vaccine neutralizing antibody responses were not impaired by concurrent asymptomatic *Plasmodium falciparum* parasitemia in HIV-infected adult Kenyans. This finding suggests that other mRNA vaccines will be immunogenic should vaccinees have subclinical malaria.

Nucleoside-modified messenger ribonucleic acid (mRNA) vaccines are a novel class of vaccines. The World Health Organization prequalified 2 COVID-19 mRNA vaccines both of which code for the SARS-CoV-2 Spike protein and are coated by lipid nanoparticles which protect the mRNA from degradation, deliver them inside cells and function as adjuvants. The remarkable success of these vaccines against severe COVID-19, and their adaptability to other microbial pathogens, holds great promise for transforming infectious disease prevention, including for HIV, malaria, and tuberculosis These global health scourges profoundly affect residents of malaria-endemic regions who are repeatedly infected by *Plasmodium falciparum* (*Pf*) from childhood and as adults commonly experience asymptomatic parasitemia, a condition facilitated by anti-protozoal immunotolerance.

A hypothesis advanced to explain low rates of severe COVID-19 in Africa was that frequent exposure to *Pf* parasitemia is protective due to immunotolerance. Evidence was found in a prospective cohort of SARS-CoV-2 infected Ugandans simultaneously tested for serologic markers of *Pf* exposure: restricted to patients without comorbidities, increasing malaria exposure was inversely associated with severe COVID-19 [1]. Some have speculated that cross-reactivity with *Pf* epitopes may reduce the severity of COVID-19 [2] but such immune hyporesponsiveness also raises concerns for poor immunogenicity against COVID-19 vaccines.

Scientists have long been concerned that vaccines may be inadequately immunogenic during or after malaria. Diminished vaccine-induced immune responses in parasitemic persons may depend on their age, degree of parasitemia, clinical versus subclinical malaria, and type of vaccine. For example, whereas measles vaccine is unaffected, responses to polysaccharide vaccines are attenuated [3]. Moreover, due to its widespread and prolonged nature, asymptomatic parasitemia may greatly exacerbate this problem. Compared with *Pf*-uninfected adults, titers of anticircumsporozoite antibodies remained lower in *Pf*-infected adults administered a recombinant protein malaria vaccine [4]. In a study of *Pf*-exposed children, subclinical malaria was linked to “atypical” memory B cells and “exhausted” CD4+ T cells [5]. This phenomenon was thought to contribute to the failure of malaria vaccines. Memory B- and T-cell exhaustion are well-described in persons living with HIV (PLWH), a population that is the focus of the present study [6]. Poor B cell responses to the influenza vaccine have been reported in PLWH with low CD4 counts [7]. The immunogenicity of mRNA vaccines in parasitemic persons remains unstudied.

To investigate the effect of *Pf*-infection on mRNA vaccines, we conducted a single site substudy of the multisite CoVPN 3008 trial [8] to assess the immunogenicity of COVID-19 mRNA booster vaccinations in PLWH with subclinical malaria from western Kenya, a region endemic for seasonal malaria.

## METHODS

The parent trial—a prospective, randomized clinical trial—was registered on ClinicalTrials.gov, NCT05168813 with a start date in December 2021 and completed in August 2024. The Kombewa site-specific protocol and amendments were approved by Kenya's Scientific and Ethics Review Unit, with written informed consent obtained from all trial participants before enrollment.

The substudy enrolled adult participants (≥18 years) who were asymptomatic for COVID-19 or malaria and living with HIV or other comorbidity associated with severe COVID-19 without exclusions for pregnancy, CD4+ T-cell count, antiretroviral therapy (ART) use, or detectable HIV viral load (VL).

Participants had previously received one or two 100-μg doses of monovalent mRNA-1273, based on whether they were baseline point-of-care SARS-CoV-2 anti-Spike (POC anti-S, Ecotest, Assure Tech) seropositive or seronegative, respectively. During pre-enrolment, participants were randomized 1:1 in a double-blind fashion to receive a booster vaccination with either the monovalent mRNA-1273 or bivalent mRNA-1273.222 (WA-1 and BA.4/5). Trial details have been published previously [8].

### Malaria Diagnosis

Malaria parasitemia detection was performed using dried blood spots collected at month 0 (enrolment “M0”) and month 1 (enrolment “M1”) and pre-enrolment for most participants (Supplementary Figure 1). *Pf* was identified via reverse transcriptase polymerase chain reaction (PCR) targeting *Pf*/pan-*Plasmodium* 18S rRNA [9].

### Antibody Immunogenicity Assay

Serum samples were collected at M0 and M1 and tested at the National Institute for Communicable Diseases, South Africa, using a vesicular stomatitis virus (VSV)-based neutralization assay, which is not affected by ART interference and produces equivalent titers to the lentiviral assay [10]. Heat-inactivated serum samples underwent 5-fold serial dilutions in 96-well plates (Corning Life Sciences) before addition of the SARS-CoV-2 pseudovirus (Nexelis Laboratories Canada). The serum–virus complexes were mixed with Vero E6 cells (American Type Culture Collection) and incubated for 20–24 h. Infection was detected via luminescence of the luciferase gene. Neutralizing titers were calculated as the reciprocal serum dilution corresponding to the 50% inhibitory dose (ID50) and 80% (ID80) levels, standardized measures that are associated with vaccine efficacy [11].

In addition to POC anti-S testing at baseline, all participants underwent M0 SARS-CoV-2 nasal swab nucleic acid amplification testing (NAAT) and anti-nucleoprotein (anti-NP, Abbott SARS-CoV-2 IgG) serology testing. Participants with a positive result from any of these tests (NAAT during the 6 months prior to or at M0, POC anti-S at baseline, or anti-NP at baseline or newly positive at M0) were considered to have evidence of prior SARS-CoV-2 infection. Since all participants received at least 1 dose of COVID-19 mRNA vaccine before enrolment into the malaria substudy, those with prior infection were classified as having “hybrid immunity,” while the remaining participants were classified as having “vaccine immunity.”

### Statistical Analyses

The analytic cohorts included participants who (a) received the booster at M0; (b) had malaria PCR test results at both M0 and M1 or were *Pf*-PCR positive at M0 and missing at M1; and (c) had their M1 neutralizing antibody titer collected within 15–42 days post-booster.

In the primary objective cohort analysis, participants were classified as *Pf*-uninfected if *Pf*-PCR was negative at both M0 and M1, regardless of earlier *Pf*-PCR results; participants with a negative *Pf*-PCR at M0 and a positive test result at M1 were excluded because their inclusion could alter the response to the booster. In secondary analyses, we considered participants positive if they were *Pf*-PCR positive at either M0 or M1 (secondary objective cohort “a”), if they were *Pf*-PCR positive 4–5 months prior to enrolment or M0 or M1 (secondary objective cohort “b”), and if they were *Pf*-PCR positive up to 6 months prior to enrolment or M0 or M1 (secondary objective cohort “c”).

We used the SARS-CoV-2 anti-D614G-Spike neutralizing antibody titer as the immunogenicity biomarker, as it is an inverse correlate of risk for symptomatic COVID-19 [12]. Titers below the lower limit of quantification (LLOQ) were imputed as half the value of LLOQ. The primary endpoints were anti-D614G-Spike-neutralization at the ID50 and ID80 levels at M0 and M1. Comparisons of proportions between 2 groups were performed using Barnard's test. Comparisons of the geometric mean neutralization antibody titers (GMT) at a single time-point, and the geometric mean M1-to-M0 fold-rise ratio (GMFR) between 2 groups were performed using *t*-tests in univariate unadjusted analyses, and in multivariate linear regression models to adjust for other covariates. Missing data were not imputed. All analyses were performed using R version 4.04 (R Foundation for Statistical Computing, Vienna, Austria).

### Malaria Substudy Sample Size

All 330 participants enrolled in CoVPN 3008 at the Kombewa site were invited to join the malaria substudy.

## RESULTS

### Participant Selection

Participants were enrolled into the substudy and received the booster between January and March 2023, coinciding with seasonal peak malaria activity at the site. As the parent trial had completed recruitment of persons uninfected with HIV at the time of study launch in Kombewa, all Kombewa participants were PLWH. A total of 22 participants were excluded from the primary objective cohort: 4 due to missing malaria tests at M1, 17 who converted from *Pf*-negative to *Pf*-positive between M0 and M1, and 1 with missing neutralizing antibody results. The analysis included the remaining 87 *Pf*-positive and 221 *Pf*-negative participants (Supplementary Figure 2). No *Plasmodium* species other than *P. falciparum* was detected.

### Participant Characteristics

Among the 308 participants analyzed for the primary objective, 70.8% were female and median age was 37 years (range 20–72). At M0, 7.8% had CD4+ T-cell counts <350 cells/mm3, 22.4% had a VL ≥40 copies/mL and 83.8% had evidence of hybrid immunity. Participants who were *Pf*-positive were more likely to have a body mass index ≤25 (88.5% vs 78.3%, *P* = .042) and more likely to have a VL ≥40 copies/mL (29.9% vs 19.5%, *P* = .0491) than *Pf*-negative participants (Supplementary Table 1).

### Neutralizing Antibody Responses to the Booster Vaccine in Parasitemic vs Nonparasitemic Participants

**Figure 1.**
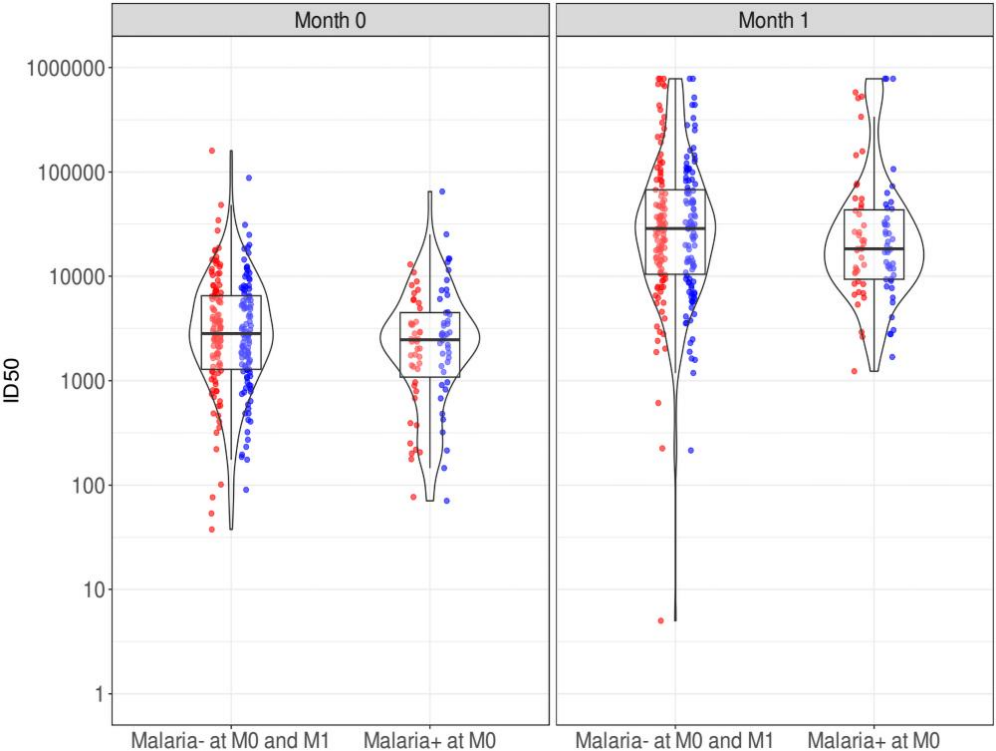
Anti-D614G Spike neutralization antibody ID50 titers to booster vaccines in asymptomatic *Plasmodium falciparum*-PCR-negative (*n* = 221) and -positive (*n* = 87) participants at Month-0 and Month-1. Box plots are superimposed on each violin plot, providing the median, 2 hinges which correspond to the first and third quartiles and “whiskers,” which extend to 1.5-times the interquartile range on both ends. Red and blue dots represent monovalent mRNA 1273 and bivalent mRNA 1273.222 booster recipients, respectively. Abbreviations: ID50, 50% inhibitory dose; M0, Month-0 when the booster dose was administered; M1, Month-1; neg, negative by PCR; pos, positive by PCR.

A statistically nonsignificant lower ID50 anti-D614G Spike neutralizing antibody titer response at M0 was observed in participants who were *Pf*-positive versus those who were *Pf*-negative, with GMT 2079 and 2765, respectively (*P* = .084, Figure 1; Supplementary Table 2). Similarly, at M1 both groups exhibited a robust booster response, GMT 22 019 and 26 932, respectively (*P* = .270).

**Figure 2.**
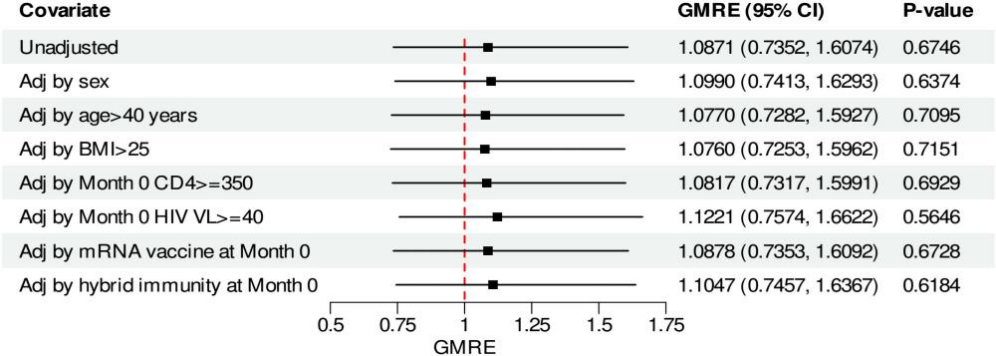
ID50 geometric mean ratio estimates of neutralizing antibody Month-1 over Month-0—when participants received a booster vaccine—comparing participants with and without asymptomatic *Plasmodium falciparum* parasitemia and showing adjustments for covariates in univariate analysis. Abbreviations: Adj, adjusted; BMI, body mass index; CD4, CD4+ T cells in cells/mm^3^ measured at Month 0; CI, confidence intervals; GMRE, geometric mean ratio estimate (a ratio of M1 over M0 of ID50 geometric mean fold-rises in parasitemic over nonparasitemic participants); ID50, 50% inhibitory dilution neutralizing anti-SARS-CoV-2 antibody titer; VL, HIV viral load in copies/mL measured at M0.

The ID50 GMFR was comparable between participants with parasitemia (10.6, 95% CI [CI] 7.6–14.8) and without parasitemia (9.7, 95% CI 7.9–12.0, Supplementary Table 3). The GMFR between *Pf*-positive and *Pf*-negative participants (defined as the geometric mean ratio estimate) was not significantly different, 1.1 (*P* = .675), and none of the following covariates significantly affected this relationship: sex, age >40 years, body mass index >25, VL ≥40 copies/mL at M0, monovalent versus bivalent booster or hybrid versus vaccine immunity at M0 (Figure 2).

### Secondary Analyses

Additional analyses conducted for ID50 and ID80 neutralizing anti-D614G-Spike antibody levels and using alternative definitions of *Plasmodium* parasitemia status rendered similar overall findings (see Supplementary Figure 3 and Supplementary Tables 4–7).

## DISCUSSION

Given the potential importance of mRNA vaccines for preventing infectious diseases in malaria-endemic settings, we sought to determine whether parasitemia adversely affected the immune correlate of protection for COVID-19 [12] following booster vaccination. In this cohort of PLWH, we found no reduction in the neutralizing anti-Spike antibody responses to a booster dose of either the monovalent mRNA-1273 or the bivalent mRNA-1273.222 vaccine, with GMTs in both parasitemic and nonparasitemic participants exceeding by 20-fold 96% vaccine efficacy levels [12]. There was a statistically and clinically nonsignificant lower level of neutralizing antibody at both M0 and M1 in persons with parasitemia. Immunogenicity was also unaffected by various potentially confounding factors, including CD4 < 350 cells/mm^3^, VL ≥40 copies/mL, and hybrid immunity at M0. In a separate analysis of the parent trial, no diminution of the neutralizing anti-Spike antibody response was observed in PLWH as compared with persons without HIV infection [13].

Studies of vaccines in parasitemic children and adults have shown mixed effects on vaccine immunogenicity [3]. The vaccines studied included recombinant protein, polysaccharide, live oral attenuated bacteria, and viral vector vaccines. Our study makes a novel contribution to this literature by demonstrating the elicitation of high titers of neutralizing antibodies by a COVID-19 mRNA vaccine in asymptomatic *Pf*-positive adults.

Our findings align with recent studies of parasitemic adults immunized with Ebola virus vaccines (rVSVΔG-ZEBOV-GP and Ad26.ZEBOV, MVA-BN-Filo regimen) and 2 recombinant protein malaria vaccines one of which immunized children with RTS,S/AS01 [14, 15]. Immune responses may vary according to specific vaccine platforms and recipient characteristics, emphasizing the need to determine actual vaccine efficacies in *Pf*-positive persons rather than relying solely on immunogenicity. In the case of COVID-19, the surrogate marker for protection—the neutralizing antibody titer—has been established and was utilized in our evaluation.

Our study has several limitations. First, repeated SARS-CoV-2 infections among participants prior to receiving the booster vaccination could have masked reductions in immunogenicity due to parasitemia. Second, a larger sample size may have improved the precision of our GMFR estimates. Third, our use of a 100 μg booster dose may have induced higher antibody responses than the currently recommended 50 μg booster dose; leaving uncertainty about the effect of the approved lower dose of the mRNA vaccine. Fourthly, parasitemia was measured qualitatively and not quantified preventing a more detailed evaluation of immune responses by parasitemic intensity. As asymptomatic parasitemic persons typically have low levels of peripheral parasitemia, this issue may not be a significant concern. Lastly, as all participants were adult PLWH our findings may not be generalizable to children or HIV-negative persons. It should be noted that PLWH with a suppressed VL and CD4 > 200 cells/mm^3^ respond similarly to vaccines as healthy adults.

In summary, among PLWH, the neutralizing antibody response to mRNA booster vaccination—the established correlate of protective immunity against symptomatic COVID-19—was comparable between those with and without subclinical malaria, supporting the further development and deployment of mRNA vaccines for other infectious diseases in malaria-endemic regions. Additional research is needed to study the immunogenicity of mRNA vaccines in parasitemic children and in persons with different degrees of parasitemia. Beyond immunogenicity, there is a long-standing need to study the efficacies of vaccines of all platforms in persons with subclinical and clinical malaria.

## Supplementary Material

jiaf398_Supplementary_Data

## Supplementary Data

Supplementary materials are available at *The Journal of Infectious Diseases* online (http://jid.oxfordjournals.org/). Supplementary materials consist of data provided by the author that are published to benefit the reader. The posted materials are not copyedited. The contents of all supplementary data are the sole responsibility of the authors. Questions or messages regarding errors should be addressed to the author.
