# Supplementary material for: Plasmodium falciparum Parasitemia Does Not Diminish Neutralizing Antibody Responses After mRNA COVID-19 Booster Vaccination in HIV-infected Adults
Source: J Infect Dis. 2025 Aug 2;232(4):e565–70. doi: 10.1093/infdis/jiaf398 (PMC12476267; doi:10.1093/infdis/jiaf398)

Supplementary Figure S1. Activities during parent study (“Pre-enrolment”) in relation to the Kombewa malaria substudy (“Enrolment”)


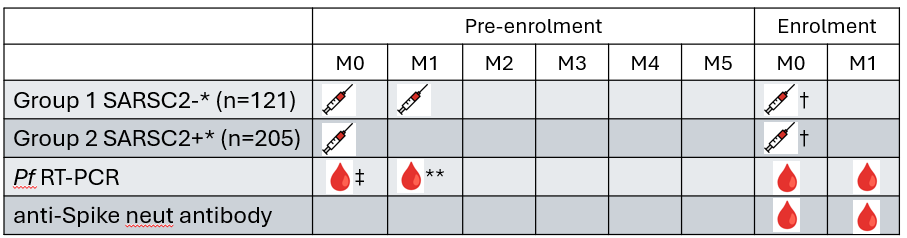


NOTE

* 4/330 participants were excluded due to missing malaria tests at Enrolment Month 0

† Randomized 1:1 at Pre-enrolment Month 0 (“baseline” in the main paper) to receive either mRNA-1273 or mRNA-1273.222 at Enrolment M0

‡ Since the parent study (Pre-enrolment period) began enrolment before the malaria substudy was IRB approved, not all participants had a Pre-enrolment M0 *P falciparum* PCR (*Pf* RT-PCR)

** Since Group 1 participants had a baseline negative point-of-care anti-SARS-Cov-2 (SARSC2) antibody test, they returned

at Pre-enrolment M1 to receive a second vaccine and were re-tested with *Pf* RT-PCR

Supplementary Figure S2. CONSORT diagram showing parent cohort and Kombewa substudy primary objective cohort


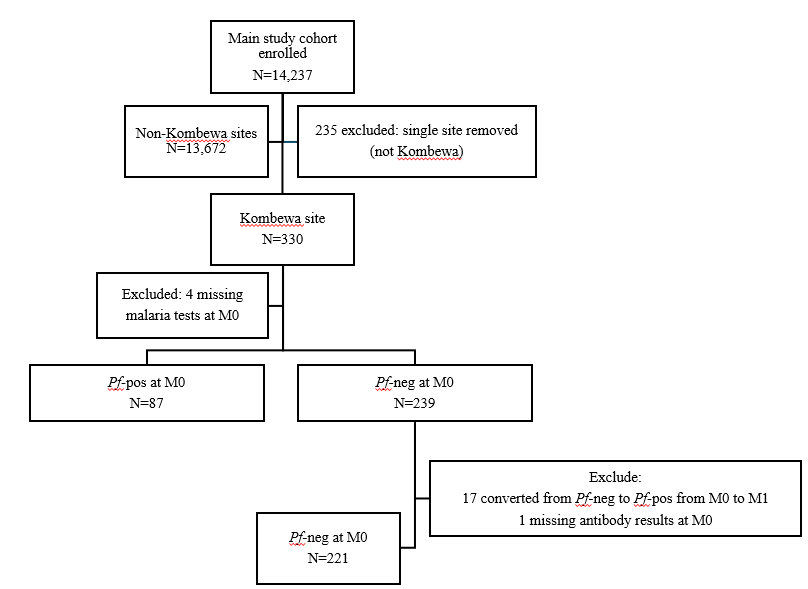


NOTE. Abbreviations. M0, enrolment into the malaria substudy; M1, 28 days after enrolment; *Pf*-neg, PCR negative for *Plasmodium falciparum*; *Pf*-pos, PCR positive for *Plasmodium falciparum*

Supplementary Table S1. Baseline characteristics at enrolment (M0) in asymptomatic *Pf*-PCR-positive (Malaria+) and -negative (Malaria-) participants using the primary objective cohort


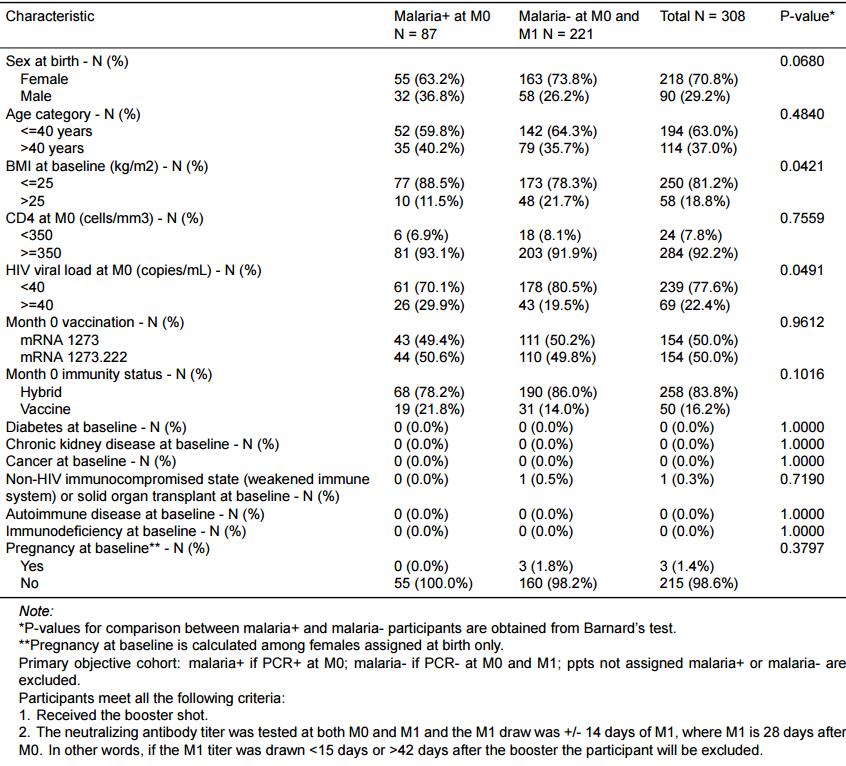


Supplementary Table S2. ID50 and ID80 geometric mean titers of neutralizing anti-D614G Spike antibody to booster vaccines at M0 (booster receipt) and M1 in asymptomatic *Pf*-PCR-positive (Malaria+) and -negative (Malaria-) participants using the primary objective cohort


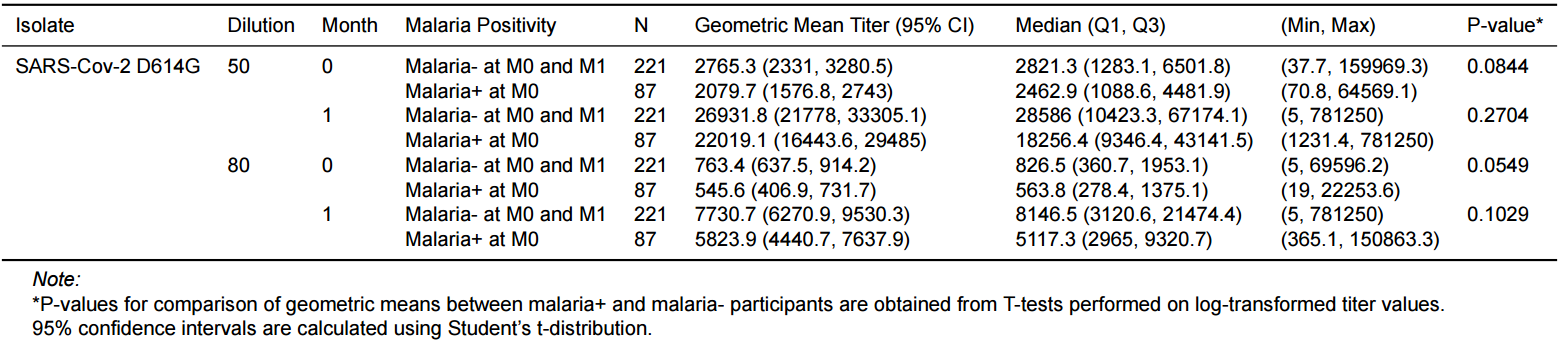


Supplementary Figure S3. Violin boxplots of ID80 neutralizing anti-D614G Spike antibody to booster vaccines in asymptomatic Pf-PCR-positive and -negative participants at M0 and M1 using the primary objective cohort (red=monovalent mRNA-1273, blue=bivalent mRNA-1273.222)


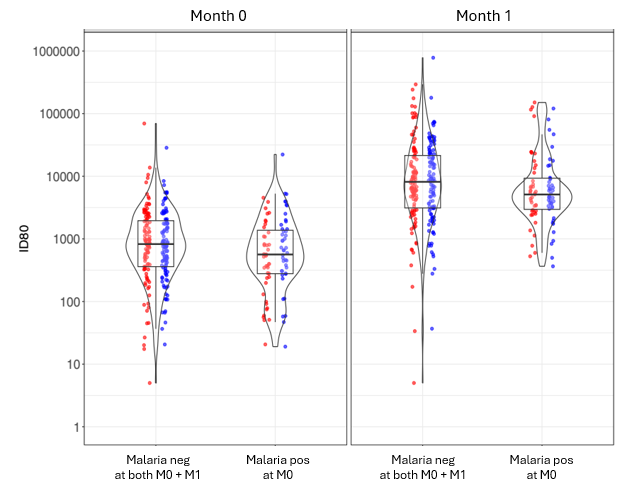


Note. Anti-D614G Spike neutralization antibody ID80 titers to booster vaccines in asymptomatic *Plasmodium falciparum*-PCR-negative (n=221) and -positive (n=87) participants at Month-0 and Month-1. Box plots are superimposed on each violin plot, providing the median, two hinges which correspond to the first and third quartiles and ‘whiskers’ which extend to 1.5-times the interquartile range on both ends. Abbreviations: ID80, 80% inhibitory dose; M0, Month-0 when the booster dose was administered; M1, Month-1; neg, negative by PCR; pos, positive by PCR.

Supplementary Table S3. ID50 and ID80 geometric mean fold rise (M1 over M0) neutralizing anti-D614G Spike antibody to booster vaccines in asymptomatic *Pf*-PCR-positive (Malaria+) and -negative (Malaria-) participants using the primary objective cohort


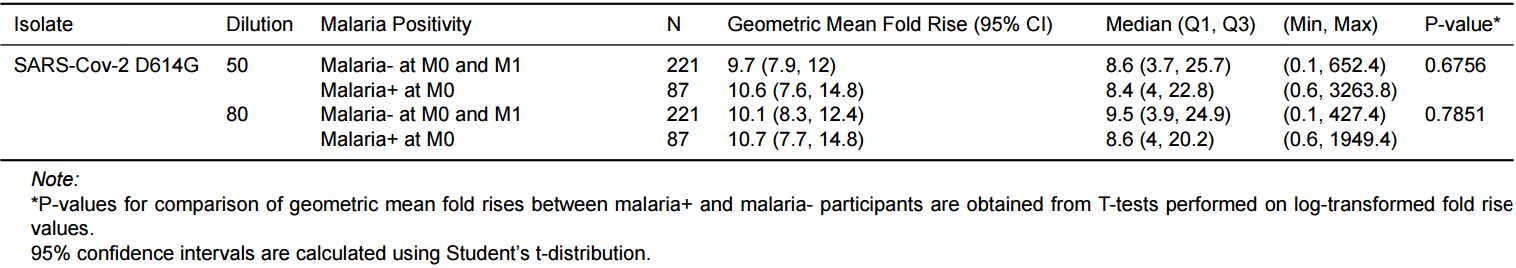


Supplementary Table S4. Base and univariate models of ID50 and ID80 geometric mean ratio estimate (M1 over M0) comparing *Pf*-PCR-positive (Malaria+) to -negative participants using the primary objective cohort (covariates measured at M0)


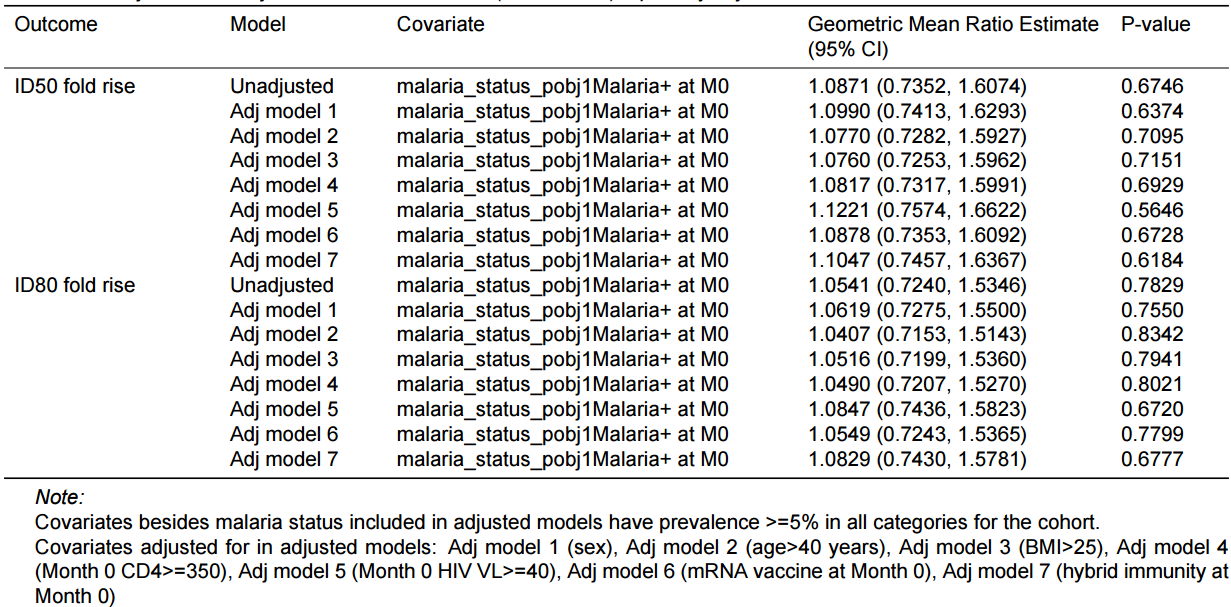


Supplementary Table S5. Base and univariate models of geometric mean ratio estimate (M1 over M0 comparing *Pf*-PCR-positive to -negative participants) using the secondary objective ‘a’ cohort (i.e., considering participants as *Pf*-PCR-positive if they tested positive at either M0 or M1; covariates measured at M0)


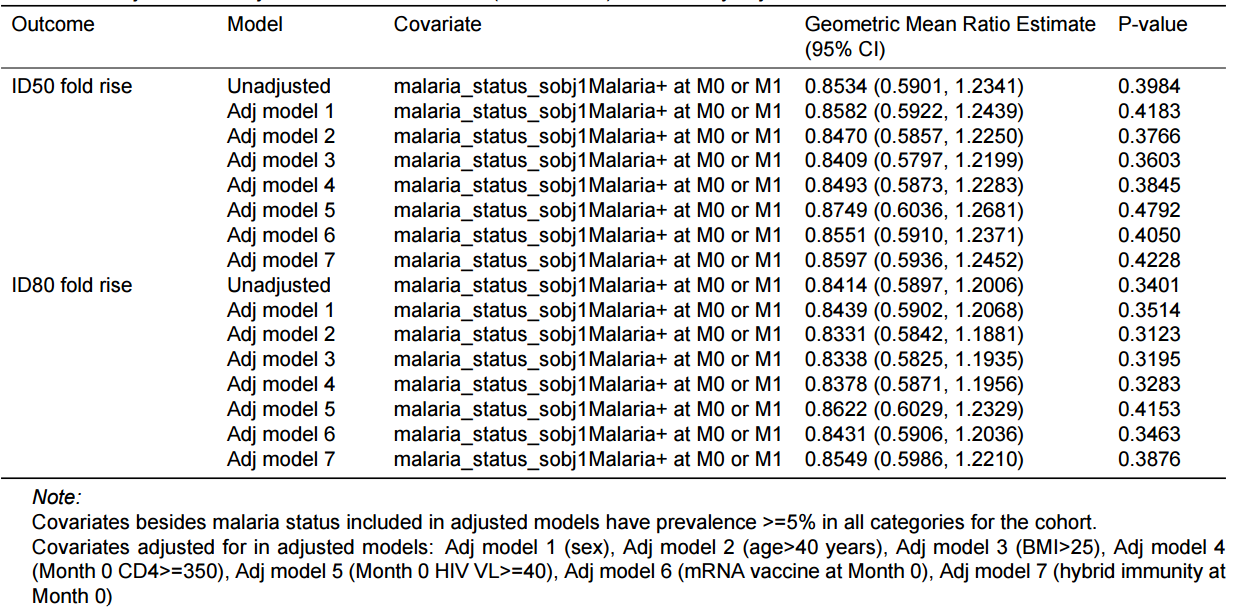


Supplementary Table S6. Base and univariate models of geometric mean ratio estimate (M1 over M0 comparing *Pf*-PCR-positive to -negative participants) using the secondary objective ‘b’ cohort (i.e., excluding *Pf*-PCR-negative participants if they had tested positive 4-5 months prior to substudy enrolment); covariates measured at M0)


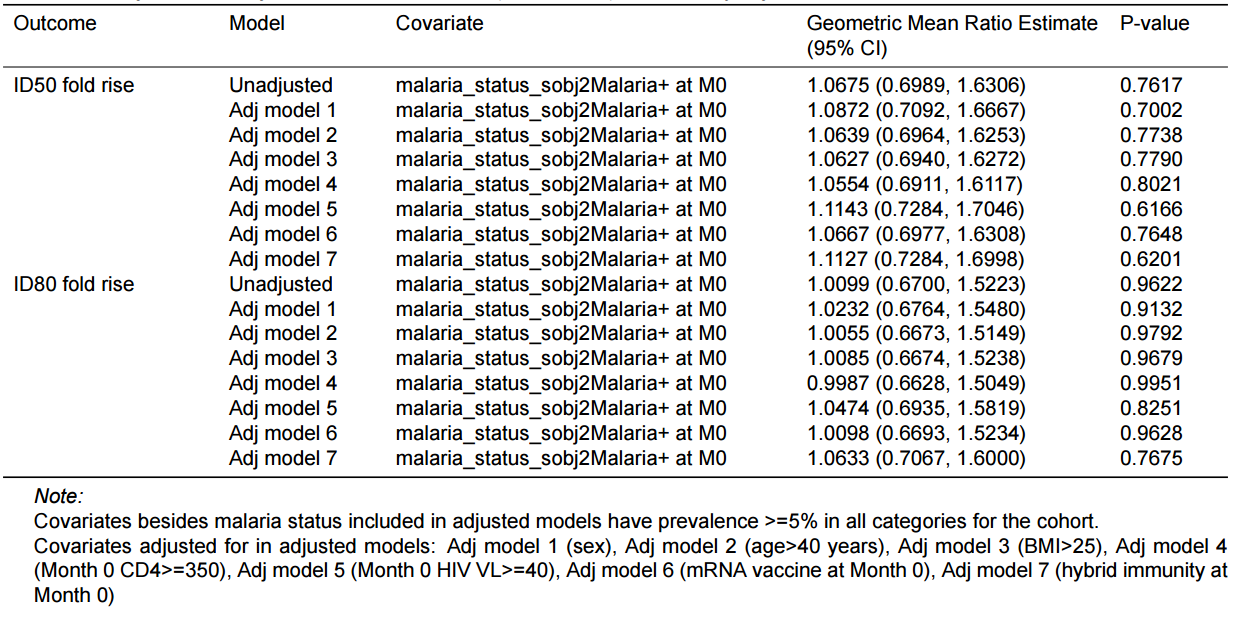


Supplementary Table S7. Base and univariate models of geometric mean ratio estimate (M1 over M0 comparing *Pf*-PCR-positive to -negative participants) using secondary objective ‘c’ cohort (i.e., excluding *Pf*-PCR-negative participants if they had tested positive up to 6 months before enrolment); covariates measured at M0)


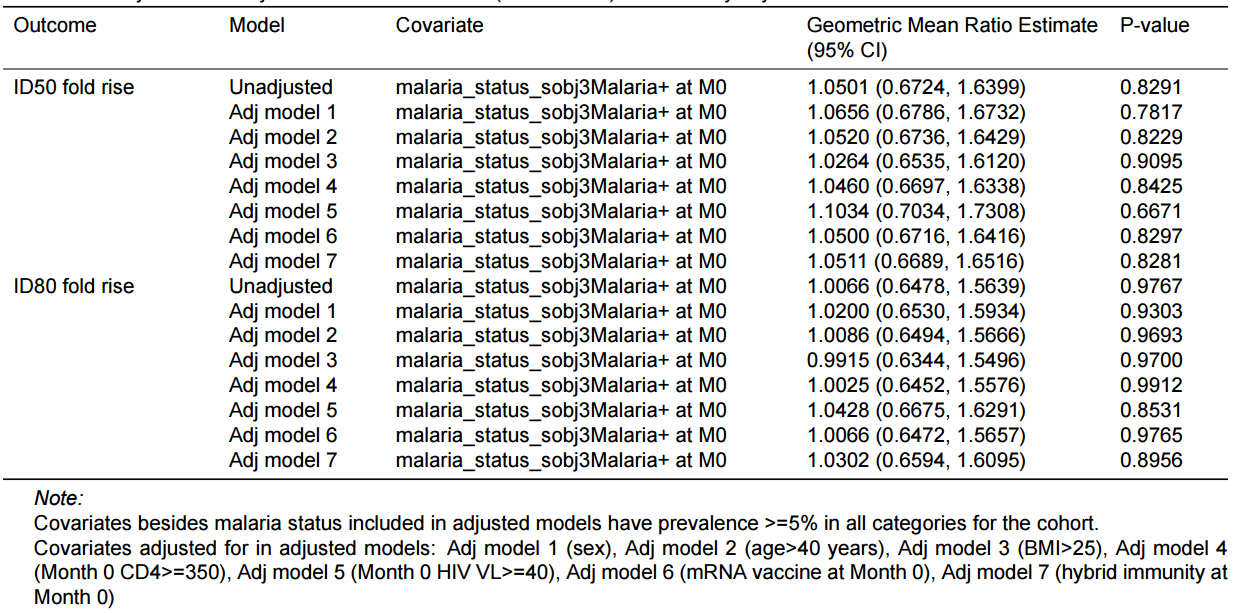

Supplement: jiaf398_Supplementary_Data [file jiaf398_supplementary_data.docx]
